# Structural re-evaluation of the human gluteus maximus

**DOI:** 10.1038/s41598-025-05361-x

**Published:** 2025-07-01

**Authors:** Hidaka Anetai, Kota Kato, Hiroyuki Kudoh, Tatsuo Sakai, Koichiro Ichimura

**Affiliations:** 1 Department of Physical Therapy, Faculty of Health Science, Juntendo University, 2-1-1 Hongo, Bunkyo-ku, Tokyo, 113-8421 Japan; 2 Department of Anatomy and Life Structure, Juntendo University Graduate School of Medicine, 2-1-1 Hongo, Bunkyo-ku, Tokyo, 113-8421 Japan

**Keywords:** Gluteus maximus, Functional anatomy, Macroscopic anatomy, Kinesiology, Hip joint, Iliotibial tract, Anatomy, Musculoskeletal system

## Abstract

The gluteus maximus (GM) is a powerful hip joint extensor acting strongly on the femur. However, most anatomical literature describes that the larger superior portion of the GM is inserted into the iliotibial tract rather than the femur, resulting in an apparent discrepancy between its structure and function. To address this, we re-evaluated the GM structure in 25 formalin-fixed Japanese cadavers. We found that the superior three-fourths of the GM formed a strong plate-like distal tendon, which was mostly inserted into the gluteal tuberosity of the femur, with partial adhesions to the iliotibial tract. The remaining inferior portion terminates at a complex involving the lateral femoral intermuscular septum, plate-like distal tendon of the superior portion, and proximal tendon of the vastus lateralis. These findings resolve the functional anatomy discrepancy of the GM: the larger superior portion is primarily inserted into the femur, contributing hip joint movement, while the smaller inferior portion supports this function. Kinesiological data on the GM should be reinterpreted based on this precise anatomy. This will significantly improve our understanding of human GM function.

**Supplementary Information:**

The online version contains supplementary material available at 10.1038/s41598-025-05361-x.

## Introduction

The gluteus maximus (GM) is the largest muscle in the gluteal region in humans. Its major function is widely regarded as hip joint extension, with an additional function in stabilizing the knee joint during extension, as noted in anatomy^1–3^ and kinesiology^4,5^ textbooks. This strong hip extensor muscle has attracted substantial interest from researchers in kinesiology and comparative anatomy, particularly regarding human bipedalism. Kinesiological analyses using electromyography, 3D motion analysis systems, and biomechanical musculoskeletal models have established the GM as a major contributor to hip joint movement^6–8^. Comparative anatomical studies, including anthropological and primate research, have demonstrated that a well-developed GM is crucial for upright posture and bipedalism, with hip joint extension, in humans^9–13^.

Many anatomical descriptions, from classical to modern textbooks and studies, state that the major superior or superficial portion of the GM is inserted into the iliotibial tract, a thickened lateral part of the fascia lata extending between the iliac crest and the lateral condyle of the tibia, stabilizing the hip and knee joints, while the minor inferior or deep portion is inserted into the gluteal tuberosity, which transitioned into the lateral lip of the linea aspera of the femur, extending the hip joint^1–3,14^. However, following these descriptions, the smaller volume portion of the GM serves its primary function (hip joint extension), whereas the larger portion serves an additional function (knee joint stabilization). The reasons for this apparent discrepancy are unclear; therefore, it warrants revisiting from the viewpoint of functional anatomy.

Recently, a kinesiological study using electromyography and ultrasound imaging demonstrated that GM’s mechanical influence on the iliotibial tract is negligible in humans^15^indicating its primary function is limited to hip joint movement. Considering not only anatomical perspectives but also such recent kinesiological evidence, we hypothesize that an inadequately explored structural relationship between the GM and iliotibial tract exists. Although most of the GM fascicles are generally considered to be inserted into the iliotibial tract, some differences exist among previous descriptions (Table 1). To what extent are the GM fascicles actually inserted into the iliotibial tract? The kinesiological study suggested that the GM contraction have no functional impact on the iliotibial tract—does this imply that the GM fascicles have only a limited attachment to the iliotibial tract? If so, where and how are they actually inserted? Alternatively, is there any structure that counteracts the functional impact of the GM contraction on the iliotibial tract? If clarified, this relationship could resolve the discrepancy and align with recent kinesiological evidence. Furthermore, as most kinesiological analyses are grounded in anatomy, our present structural re-evaluation of the GM could be a fundamental yet crucial step in understanding its functional anatomy.

**Table 1:** Classical to modern anatomical descriptions of the human GM.

Authors	Book title	Descriptions		
		Division	Origin	Insertion*
Browne^23^	A Complet Treatise of the Muscles, As They Appear in Humane Body, and Arise in Dissection	−	Ilium, sacrum	Linea aspera
Winslow^24^	Exposition Anatomique de la Structure du Corps Humain	−	Ilium, sacrum, coccyx	Linea aspera, fascia lata
Cowper^25^	The Anatomy of the Humane Bodies	−	Ilium, sacrum, coccyx, sacrotuberous ligament	Linea aspera, fascia lata
Albinus^26^	Tabulae Sceleti et Musculorum Corporis Humani	−	Ilium, sacrum, coccyx, thoracolumbar fascia,	Linea aspera, vastus lateralis
Sabatier^27^	Traité Complet D’Anatomie ou Description de Toutes les Parties du Corps Humain	−	Ilium, sacrum, coccyx, posterior sacroiliac ligament	Linea aspera, fascia lata, vastus lateralis
Cheselden^28^	The Anatomy of the Human Body	−	Ilium, sacrum, sacrotuberous ligament	Linea aspera
Bell^29^	The Anatomy of the Human Body (4th ed.)	−	Ilium, sacroiliac joint, sacrum, sacrotuberous ligament	Root of the great trochanter, linea aspera
Bock^30^	Handbuch der Anatomie des Menschen	−	Ilium, sacrum, coccyx, posterior sacroiliac ligament	Linea aspera, fascia lata
Hyrtl^31^	Lehrbuch der Anatomie des Menschen mit Rücksicht auf physiologische Begründung und praktische Anwendung	−	Coccyx, thoracolumbar fascia, sacrotuberous ligament	Linea aspera, greater trochanter, fascia lata
Quain et al.^32^	Human Anatomy	Upper portion (larger)	Ilium, sacrum, posterior sacroiliac ligament, sacrotuberous ligament, coccyx	Fascia lata
		Lower portion		Gluteal tuberosity
Gegenbaur^33^	Lehrbuch der Anatomie des Menschen	Upper portion	Ilium, sacrum, sacrotuberous ligament	Fascia lata
		Lower portion		Gluteal tuberosity
Merkel^34^	J. Henle’s Grundriss der Anatomie des Menschen	Superficial portion	Ilium, sacrum, thoracolumbar fascia, coccyx	Fascia lata
		Deep portion	Ilium, sacrum, sacrotuberous ligament	Linea aspera
Testut^42^	Traité D’ Anatomie Humaine	Upper portion	Ilium, posterior sacroiliac ligament, thoracolumbar fascia, sacrum, coccyx, sacrotuberous ligament	Gluteal tuberosity
		Lower portion		Fascia lata
Poirier and Charpy^43^	Traité D’ Anatomie Humaine	Superficial portion	Ilium, thoracolumabar fascia, sacrum, coccyx, poserior sacroiliac ligament, gluteal aponeurosis	Fascia lata
		deep portion		Linea aspera
Gray^35^	Anatomy of the Human Body (20th ed.)	Upper portion & superficial fibers of lower portion	Ilium, sacrum, coccyx, thoracolumbar fascia, sacrotuberous ligament, gluteal aponeurosis	Iliotibial tract (of fascia lata)
		Deep fibers of lower portion		Gluteal tuberosity
Piersol^36^	Human Anatomy (7th ed.)	Upper portion	Ilium, sacrum, coccyx, posterior sacroiliac ligament, sacrotuberous ligament	Iliotibial tract (of fascia lata)
		Lower portion		Gluteal tuberosity
Kopsch^37^	RAUBER/KOPSCH Lehrbuch und Atlas der Anatomie des Menschen	Upper two thirds portion	Ilium, thoracolumbar fascia, sacrum, coccyx, sacrotuberous ligament	Fascia lata
		Lower third portion		Gluteal tuberosity
Schaeffer^2^	Morris’ Human Anatomy: A Complete Systematic Treatise (10th ed.)	Large superficial portion	Ilium, thoracolumbar fascia	Iliotibial tract
		Deep portion	Sacrum, coccyx, sacrotuberous ligament	Gluteal tuberosity, vastus lateralis origin
Williams^3^	Gray’s Anatomy (38th ed.)	Upper portion & superficial fibers of lower portion	Ilium, thoracolumbar fascia, sacrum, coccyx, sacrotuberous ligament, gluteal aponeurosis	Iliotibial tract
		Deep fibers of lower portion		Gluteal tuberosity
Rosse et al.^38^	Hollinshead’s Textbook of Anatomy	Upper portion & superficial fibers of lower portion	Ilium, sacrum, coccyx, sacrotuberous ligament	Iliotibial tract
		Deep fibers of lower portion		Gluteal tuberosity
Rouvière and Delmas^44^	Anatomie Humaine: Descriptive, Topobraphique et Fonctionnelle (15th ed.)	Superficial portion	Ilium, sacrum, coccyx, sacrotuberous ligament, gluteal aponeurosis	Fascia lata
		Deep portion		Linea aspera, lateral intermuscular septum
Paulsen and Waschke^39^	Sobotta Atlas of Human Anatomy (15th ed.)	Superficial portion	Sacrum, ilium, thoracolumbar fascia	Iliotibial tract, fascia lata
		Deep portion	Sacrotuberous ligament	Gluteal tuberosity
Gilroy et al.^40^	Atlas of Anatomy (2ed ed.)	Upper portion	Sacrum, ilium, thoracolumbar fascia, sacrotuberous ligament	Iliotibial tract
		Lower portion		Gluteal tuberosity
Koshi^1^	Cunningham’s Manual of Practical Anatomy (16th ed.)	Superficial three quarters portion	Ilium, sacrum, coccyx, sacrotuberous ligament	Iliotibial tract
		Deep quarter portion		Gluteal tuberosity
Moore et al.^41^	Clinically Oriented Anatomy (8th ed.)	Superior portion & superficial fibers of inferior portion	Ilium, sacrum, coccyx, sacrotuberous ligament	Iliotibial tract
		Deep fibers of inferior portion		Gluteal tuberosity

*The iliotibial tract is a thickened lateral part of the fascia lata; the gluteal tuberosity transitions into the linea aspera (lateral lip) on the posterior surface of the femur.

In this study, we aim to resolve the discrepancy between the anatomy and function of the human GM by re-evaluating its structure and to consider its functional anatomy. However, this may not have been fully addressed by traditional in situ dissection. We therefore re-evaluated the GM structure by combining in situ observations—including the method in which all lower-limb muscles except for the GM were removed—with supplemental observations of isolated GM specimens. Previous studies using such specimens have provided valuable insights into muscle structure and function^16–22^; thus, this combined approach may be effective for detailed evaluation of muscle ends. In addition, the precise attachment sites of the muscles, including structures in their origin and insertion regions, can also be confirmed through the isolation process.

## Results

In our structural re-evaluation, we delineated two distinct portions of the human GM based on structural differences in the distal region:

1. The superior portion is mostly inserted into the gluteal tuberosity of the femur via a strong tendon.
2. The inferior portion terminates at the complex formed by the lateral femoral intermuscular septum (LFIS), distal tendon of the superior portion, and proximal origin of the vastus lateralis.

No remarkable structural differences were observed in GM’s origin, insertion, muscle-tendon structure, and proximal and distal regions based on individual or sex. The following sections describe our findings in detail.

### Observations of superficial and deep aspects of the GM

**Fig. 1:**
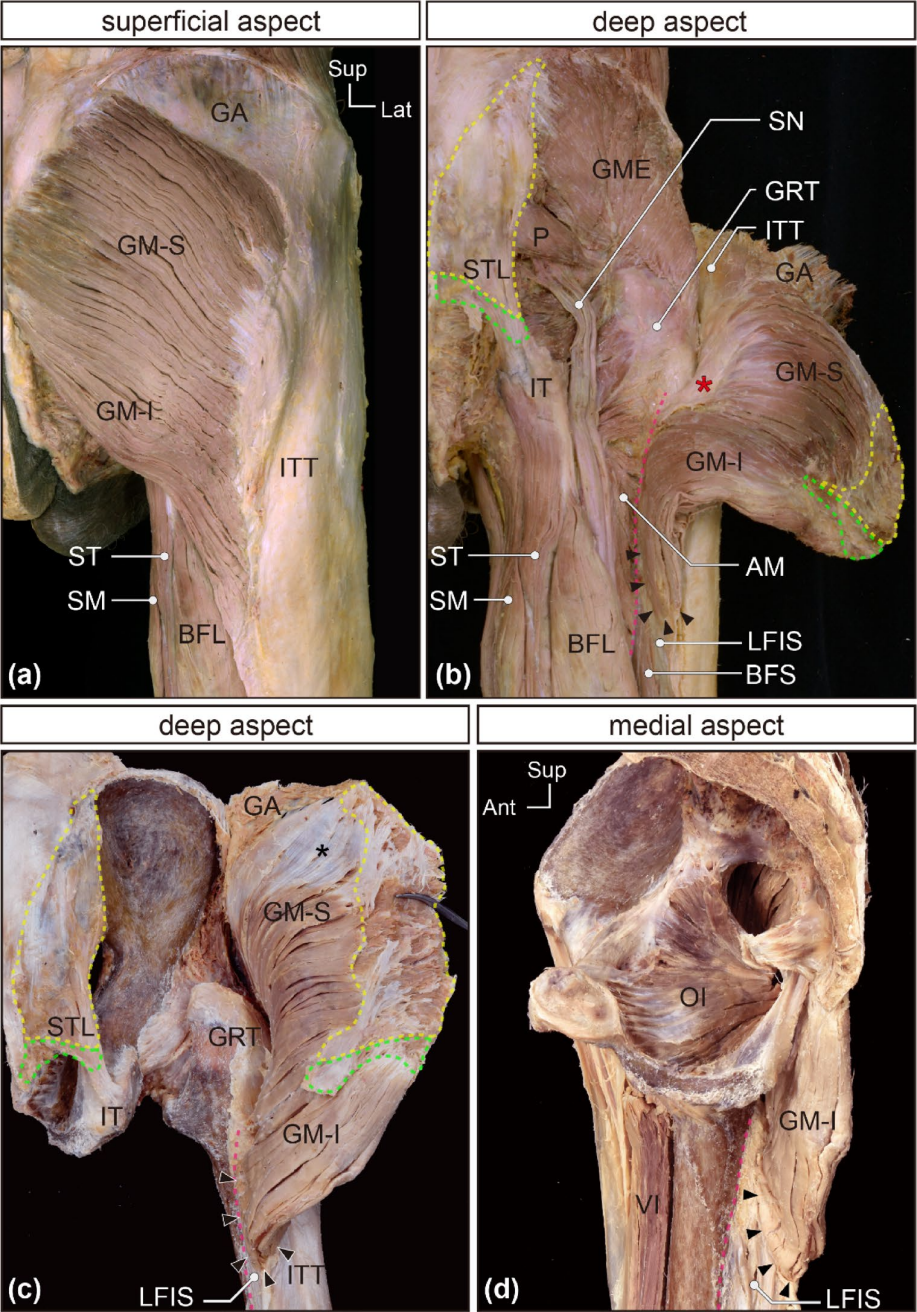
In situ observations of the GM. Photographs of a representative in situ specimen (right posterior pelvic and thigh regions) are shown. (**a**) The superficial surface of the gluteus maximus (GM) and surrounding structures are visible. (**b**) The proximal origin of the GM was detached and laterally reversed, enabling us to confirm deep surface of the GM. Additionally, an in situ specimen in which all the muscles are located in the gluteal, posterior, and medial thigh regions were removed, except for the GM and part of the quadriceps femoris, is shown. (**c**) The deep surface of the GM is visible. (**d**) The medial aspect of the inferior portion of the GM (GM-I) and the lateral intermuscular septum (LFIS) can be observed. The red asterisk marks the distal tendon of the superior portion of the GM (GM-S). The yellow dotted lines (areas) in the posterior pelvic region and deep surface of the proximal end of the GM indicate the proximal attachment site of the GM-S, and both correspond to each other. The light-green dotted lines (areas) indicate the proximal attachment site of the GM-I. Black asterisk marks the proximal tendon located in the uppermost part of the deep surface of the GM. Black arrowheads indicate the attachment site of the GM-I to the LFIS. The magenta dotted lines trace the gluteal tuberosity and the lateral lip of the linea aspera, which are continuous. AM, adductor magnus; BFL, long head of the biceps femoris; BFS, short head of the biceps femoris; GA, gluteal aponeurosis; GM, gluteus maximus; GM-I, inferior portion of the GM; GME, gluteus medius; GM-S, superior portion of the GM; GRT, greater trochanter; IT, ischial tuberosity; ITT, iliotibial tract; LFIS, lateral femoral intermuscular septum; OI, obturator internus; P, piriformis; SM, semimembranosus; SN, sciatic nerve; ST, semitendinosus; STL, sacrotuberous ligament; VI, vastus intermedius.

Upon removal of the gluteal fascia, the muscle fascicles on the superficial surface of the GM were observed to run inferolaterally and then form the distal tendon, which immediately appeared to be merged and inserted into the iliotibial tract (Fig. 1a). The iliotibial tract, located in the superficial lateral thigh region as a thickened part of the fascia lata, extended between the iliac crest and the lateral condyle of the tibia. This was fixed to the lateral lip of the linea aspera, lateral supracondylar line, and lateral epicondyle of the femur by the LFIS. Upon detaching the GM origin from the bony pelvis and reflecting the proximal half of the GM to observe its deep surface, the muscle fascicles visible on the deep surface appeared to attach to the gluteal tuberosity via a distal tendon (red asterisk in Fig. 1b) and directly to the LFIS (black arrowheads in Fig. 1b–d). The fascicles of the superior portion of the GM were arranged in series with the fibers of the distal tendon, transitioning into it. Conversely, the fascicles of the inferior part directly attached to the LFIS from its medial side, with no transitional relation observed between the two.

### Divisions of the GM

Based on these structural differences in the distal regions, the GM was distinctly divided into superior and inferior portions. Upon carefully separating the superficial surface of the GM from the iliotibial tract, while avoiding transversely cutting the tendinous fibers as much as possible, it was clearer that the superior GM fascicles transitioned serially into a plate-like distal tendon. This transition pattern was observed on both the superficial and deep surfaces of the superior portion. The plate-like distal tendon gradually converged and ran deep, partially adhering to the deep aspect of the iliotibial tract (Fig. 1a and b). The inferior fascicles attached muscularly to the LFIS and a small superficial part of the iliotibial tract, rather than to the femur; that is, the distal tendon was macroscopically invisible (black arrowheads in Fig. 1b). In situ specimens, in which all muscles were removed from the gluteal, posterior, and medial thigh regions except for the GM and part of the quadriceps femoris, showed that the inferior fascicles had no attachment to the femur (black arrowheads in Fig. 1c and d).

### Anatomy of the superior portion

Approximately superior three-quarters of the GM fascicles originated broadly from the posterior end of the iliac crest, thoracolumbar fascia, sacrum, posterior sacroiliac ligament, and approximately the upper half of the sacrotuberous ligament (areas indicated by yellow dotted lines in Fig. 1b and c). The uppermost deep region fascicles originated via a proximal tendon (black asterisk in Fig. 1c), while the rest originated muscularly.

**Fig. 2:**
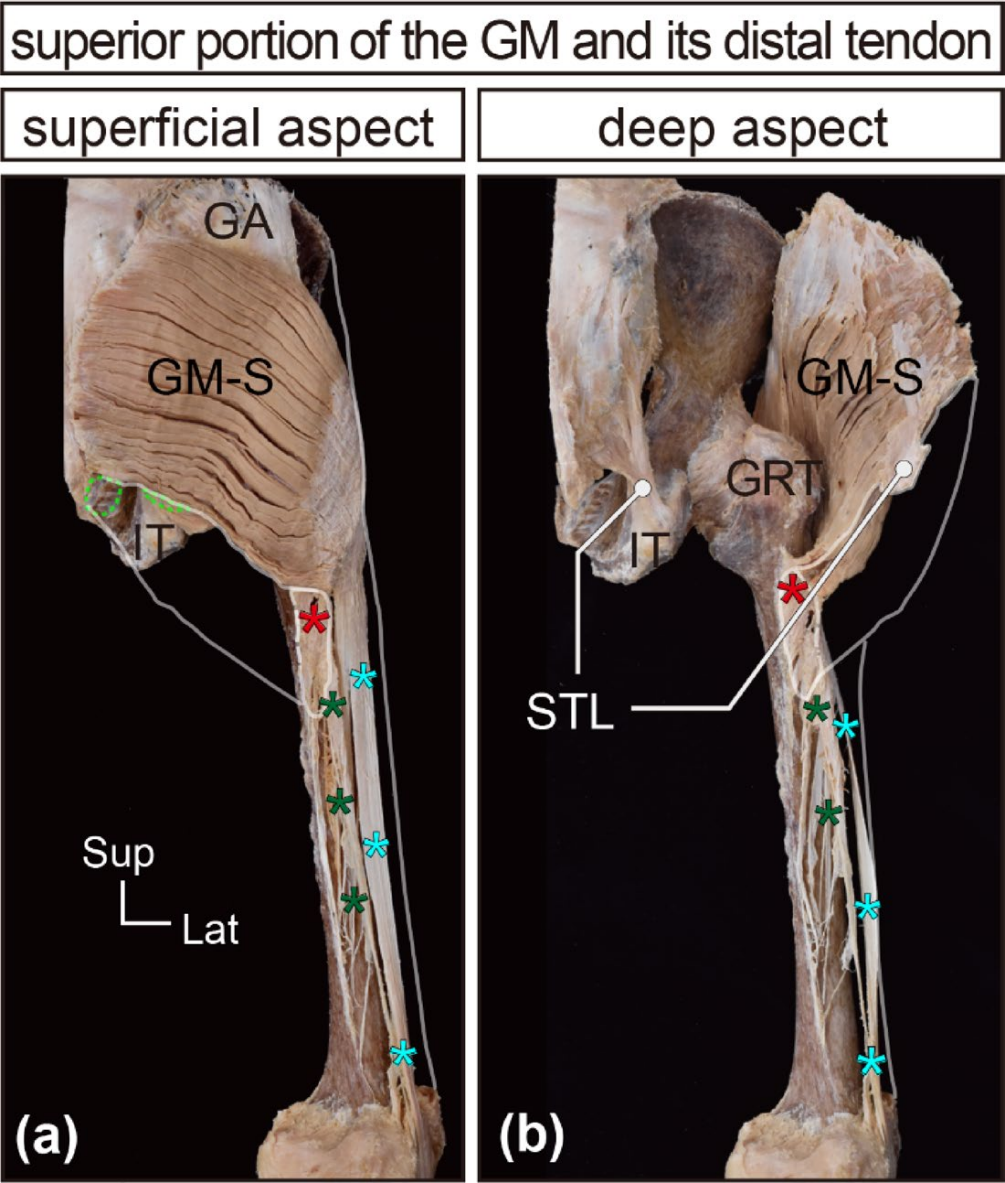
In situ observations of the superior portion of the GM (further evaluation). A representative case for further evaluation of in situ gluteus maximus (GM) specimens is presented. In (**a**) and (**b**), all pelvic, medial, and posterior thigh muscles, the inferior portion of the GM (GM-I), iliotibial tract (ITT), and its insertion into the shaft of the femur (lateral intermuscular septum, LFIS) were removed, although the accessory cords from the superior portion that are united with the iliotibial tract and LFIS remain in situ (indicated by light-blue and green asterisks, respectively). The red asterisks mark the distal tendon of the superior portion of the GM (GM-S). The light-green dotted lines (areas) in the posterior pelvic region indicate the proximal attachment site of the GM-I. The whitish lines indicate the original location of the GM-I and ITT in the in situ specimen. GA, gluteal aponeurosis; GM, gluteus maximus; GM-I, inferior part of the gluteus maximus; GM-S, superior part of the gluteus maximus; GRT, greater trochanter; IT, ischial tuberosity; ITT, iliotibial tract; STL, sacrotuberous ligament.

**Fig. 3:**
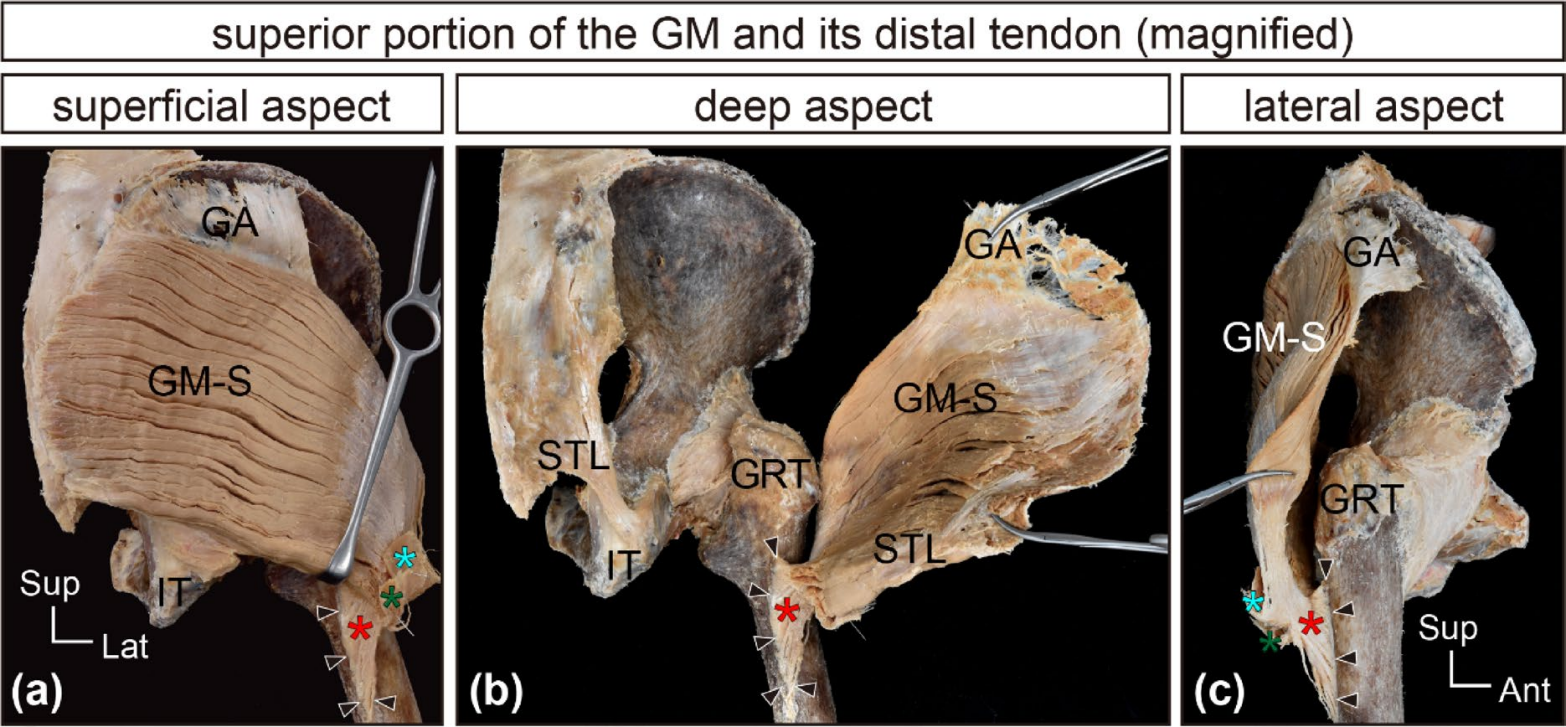
Distal tendon of the superior portion of the GM (magnified). A representative case for further evaluation of in situ superior portion of the gluteus maximus (GM-S) specimens and its distal tendon is presented. In (**a**), (**b**), and (**c**), to visualize the entire structure of the distal tendon from three angles, the GM-S and its distal tendon were focused on, and the two types of accessory cords were severed and inverted superiorly (indicated by light-blue and green asterisks). The red asterisks mark the distal tendon of the GM-S. Black arrowheads indicate the attachment site of the distal tendon of the GM-S to the gluteal tuberosity of the femur. GA, gluteal aponeurosis; GM, gluteus maximus; GM-S, superior part of the gluteus maximus; GRT, greater trochanter; IT, ischial tuberosity; STL, sacrotuberous ligament.

**Fig. 4:**
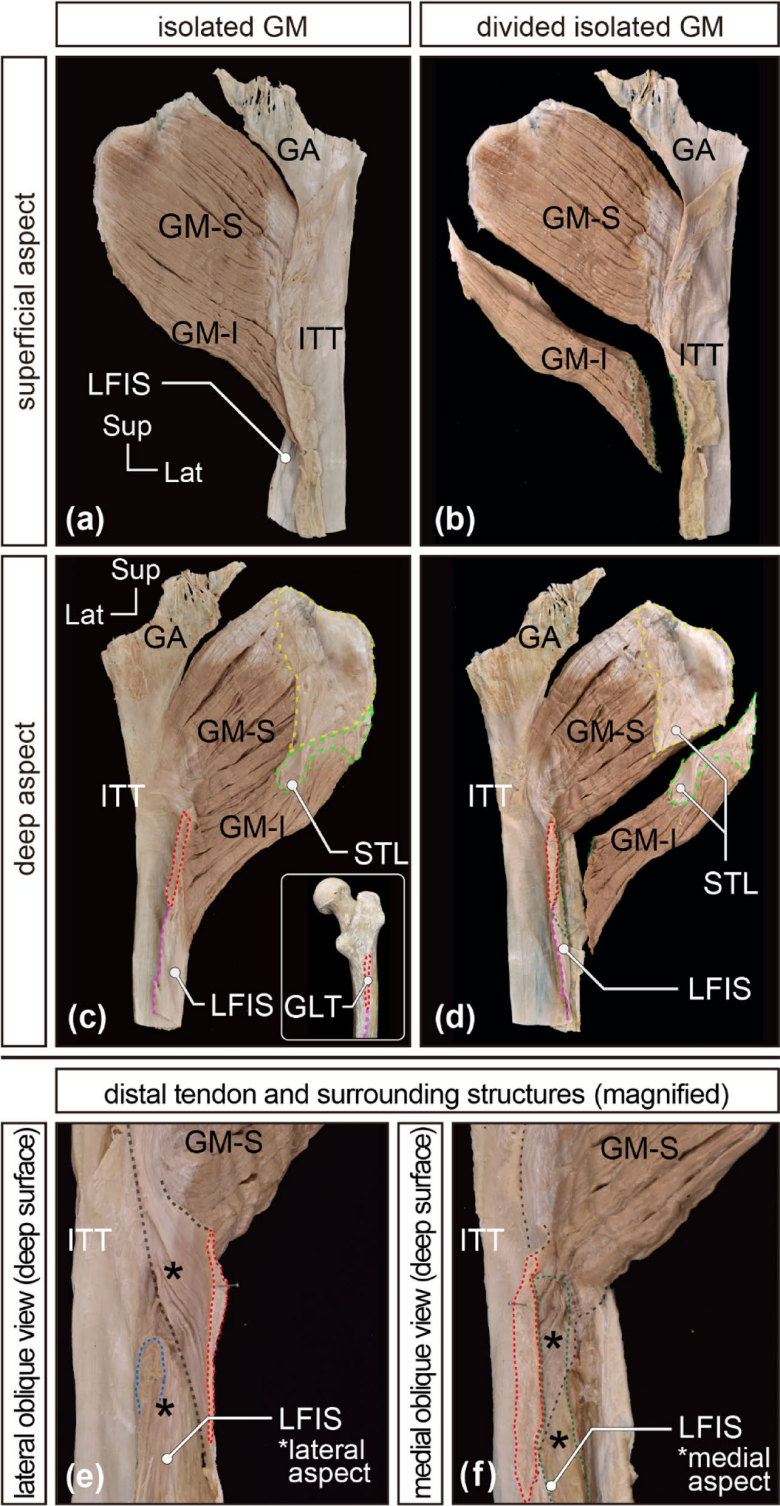
Observations of the isolated GM specimen. Representative cases of the isolated gluteus maximus (GM) specimens are arranged to show the entire views and magnified details. The entire isolated GM specimen is shown from two aspects: superficial (**a** and **b**) and deep (**c** and **d**). The isolated GM specimens indicated in (**b**) and (**d**) were demarcated into the superior portion (GM-S) and inferior portions (GM-I). (**e**) and (**f**) are magnified details of the distal tendon of the GM-S and surrounding structures. Red dotted lines (areas) indicate the attachment site of the distal tendon of the GM-S to the GLT of the femur. Black dotted lines trace the contours of the distal tendon of the GM-S. Magenta dotted line trace the attachment site of the LFIS to the lateral lip of the linea aspera. Green dotted lines (areas) indicate the distal attachment site of the GM-I. The blue dotted lines (area) indicates the attachment site of the vastus lateralis proximal tendon. The black asterisks indicate the complex of the distal tendon of the GM-S, LFIS, and the origin tendon of the vastus lateralis. The yellow dotted lines (areas) indicate the proximal attachment site of the GM-S, while the light-green dotted lines (areas) indicate that of the GM-I. GA, gluteal aponeurosis; GLT, gluteal tuberosity; GM, gluteus maximus; GM-I, inferior part of the gluteus maximus; GM-S, superior part of the gluteus maximus; ITT, iliotibial tract; LFIS, lateral femoral intermuscular septum; STL, sacrotuberous ligament.

After removing the inferior fascicles attached to the LFIS, the iliotibial tract, and its insertion into the shaft of the femur (LFIS), the distal tendon of the GM, inserted into the femur, was clearly visible (Fig. 2). The superior portion of the GM fascicles transitioned into tendinous fibers forming a thick, plate-like distal tendon, which descended posterior to the greater trochanter with a mild curve, contacted the trochanteric bursa, twisted, and was inserted into the gluteal tuberosity (Figs. 2 and 3). The entire morphology of the distal tendon could be confirmed on the deep surface of the isolated specimens without deformation (Fig. 4). In comparison to the broad origin, the insertion area was relatively narrow. Additionally, the distal tendon united with the upper part of the LFIS and the proximal tendon of the vastus lateralis, forming a complex (Fig. 4e and f) that served as the attachment site for inferior fascicles (described precisely below).

Upon carefully tracing the tendinous fibers of the individual fascicles in the superior portion, most of these fibers joined the thick plate-like tendon (refer to Supplementary Fig. S1), which was approximately 88.2 ± 3.7% of the superior portion. The remaining small tendinous fibers adhered to the iliotibial tract. A small number of tendinous fibers derived from the uppermost superficial region of the GM adhered to the iliotibial tract, forming a thin sheet-like tendon included in the LFIS (green asterisks in Figs. 2 and 3) and also joined the deep aspect of the iliotibial tract as a thick, long band-like tendon (light blue asterisks in Figs. 2 and 3). The band-like tendon extended to the lateral epicondyle of the femur and the tibia.

### Anatomy of the inferior portion

The remaining approximately inferior one-fourth of the GM originated narrowly from the lower end of the sacrum, coccyx, and the smaller portion of approximately the middle to lower part of the sacrotuberous ligament (areas indicated by green dotted lines in Figs. 1b and c, 2a and 4c, and d). These fascicles did not transition into tendinous fibers like those in the distal tendon of the superior portion; that is, they appeared to attach directly to the medial surface of the complex formed by the thick plate-like tendon, LFIS, proximal tendon of the vastus lateralis, and extremely small superficial part of the iliotibial tract (areas indicated by green dotted lines in Fig. 4). Some small tendinous arches for blood vessels were observed at the distal attachment site. In comparison to the narrow origin, the insertion area was relatively broad.

### Quantitative data of the superior and inferior portions

**Fig. 5:**
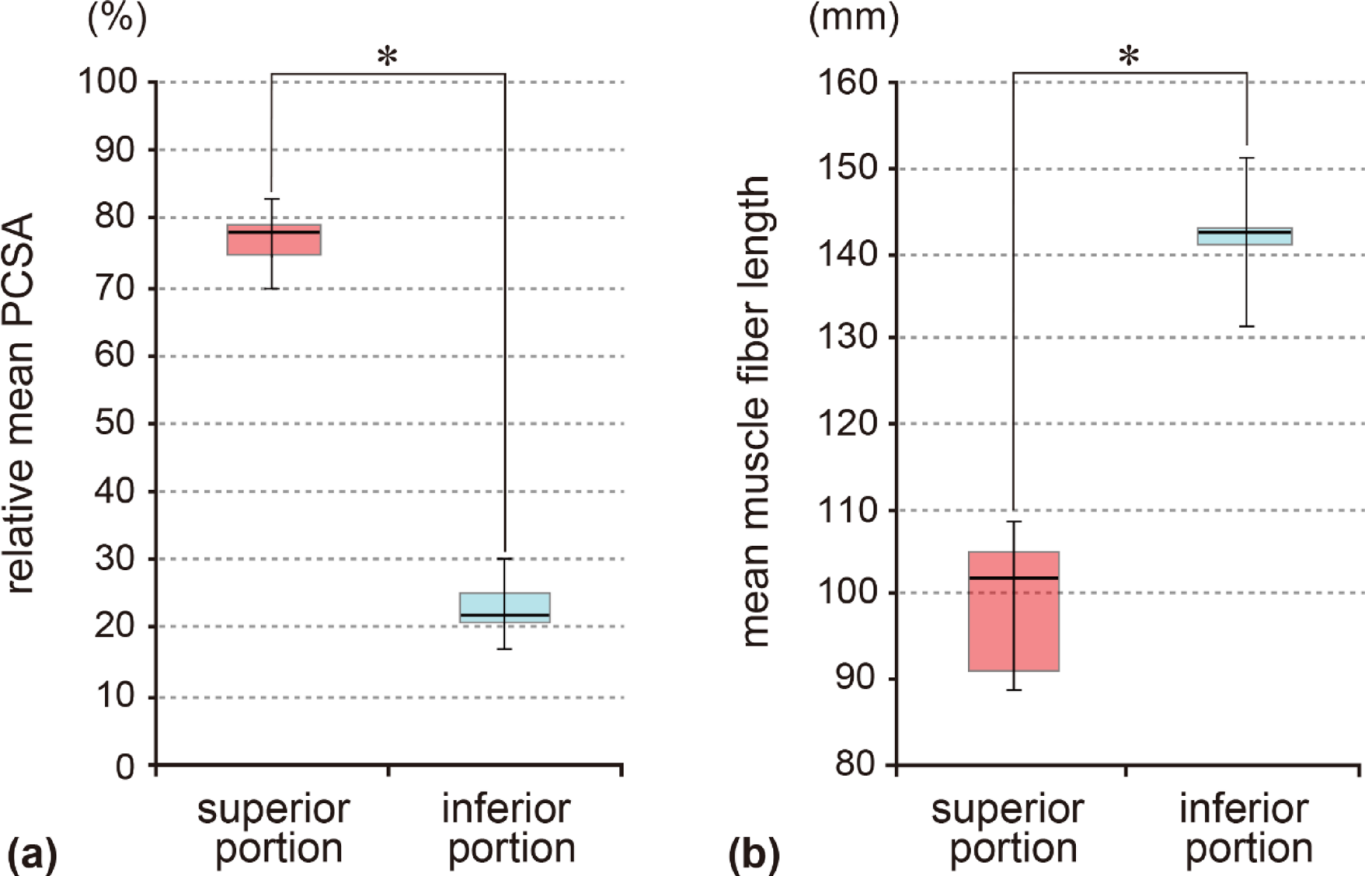
Quantitative parameters of the two portions of the GM. The human GM comprises superior and inferior portions. Box plots show the differences in the relative mean PCSA (**a**) and mean muscle fiber length (**b**) between the two GM portions (*n* = 9). Colored boxes show the values in the first to third quartiles, with the horizontal lines denoting the median. The vertical lines (whiskers) indicate the maximum and minimum values in the dataset. Differences were tested using Student’s t-tests. Statistical significance was set at *P* < 0.05.

The physiological cross-sectional areas (PCSAs) and muscle fiber lengths of the superior and inferior portions of the GM were evaluated in nine isolated GM specimens. The mean PCSAs were calculated to be as follows: 191.96 ± 43.27 mm^2^ in the superior portion, 56.54 ± 8.60 mm^2^ in the inferior portion, and 248.50 ± 46.63 mm^2^ in the whole. The relative mean PCSAs were 76.77 ± 4.27% in the superior portion and 23.23 ± 4.27% in the inferior portions. The mean muscle fiber lengths were 99.1 ± 7.79 mm in the superior portion and 142.08 ± 5.55 mm in the inferior portion. There was a statistically significant difference in the relative mean PCSA and mean muscle fiber length between the two groups (Fig. 5).

## Discussion

In this study, we re-evaluated the anatomical structure of the human GM, updating information on its insertion. The large superior portion of the GM was mostly inserted into the gluteal tuberosity via the thick plate-like tendon, and the small inferior portion was inserted into the complex including the thick plate-like tendon, LFIS, and proximal tendon of the vastus lateralis. The GM superior portion can generate a large muscle force because of its significantly larger PCSA. Conversely, the inferior portion has a significantly smaller PCSA, although it had good muscle contractility, as evidenced by its significantly longer muscle fiber length, implying that more sarcomeres are arranged per muscle fiber.

Historically, American, British, and German literature have distinguished the GM into superior (large) and inferior portions based on structural differences in the distal region, whether the fascicles were inserted into the iliotibial tract (or the fascia lata) or the femur, since the 19th century^1–3,23−41^ (Table 1). From around 1900, there was a tendency to distinguish GM compartments as superficial (inserting into the iliotibial tract) and deep (inserting into the femur). Current anatomical descriptions incorporate these criteria for distinguishing GM portions. However, inconsistency exists: the deep fibers of the inferior portion are typically described as attaching to the femur, this contrasts with both some existing descriptions and our findings. In French literature, Testut (1896) described that the inferior fascicles attach mostly to the fascia lata, with the remaining fascicles terminating on the gluteal tuberosity of the femur^42^. His description appears similar to our present findings; however, the key structure—the distal tendon terminating on the gluteal tuberosity—was not described and shown in any figure, and the transitional relationship between the muscle fascicles and tendinous fibers was not mentioned. Poirier and Charpy (1901) partially illustrated the actual structure of the distal GM tendon with accurate drawings, unique to previous reports, although this tendon was recognized as terminating at the fascia lata^43^. In their in situ observation, the distal tendon could not be pursued to its actual insertion site. In recent French anatomy texts, such as Rouvière and Delmas (2002), such descriptions not found in anatomy texts in English- or German-speaking countries have been eliminated^44^.

The reason for this misunderstanding regarding the insertion of the human GM is unclear, although partial adherence of the superior portion to the iliotibial tract is a major contributing factor. Additionally, from all the descriptions and figures in each previous report, it is understandable that GM distal end is not pursued adequately in in situ observation, which may create long-standing misconceptions regarding GM insertion and division. The present structural re-evaluation based on in situ and isolated muscle specimen analyses enabled us to thoroughly examine the muscular insertion, including the tendon, as well as the structural relationship between the muscular and tendinous fibers. The viewpoint and definition, based on the differences observed in the relationship between these fibers, contribute to structurally characterizing the distal region of the human GM, providing functional anatomical suggestions, and establishing the division of the GM into the large superior and small inferior portions.

Developmental anatomy perspectives may help to interpret the structural differences, including the insertion region and whole shape, between the superior and inferior portions in the human GM. Tický and Grim (1985) reported that the human GM comprised two fused fetal muscles: the fetal GM proper (superior) and the fetal coccygeofemoralis (inferior), which are constantly observed in human embryos and fetuses^45^. Shiraishi et al. (2018) also found that the GM is originally divided into superior and inferior portions in the early fetal period, with the inferior portion developing approximately 1 week later than the superior portion, and the two portions unite to form an adult form^46^. If the superior and inferior portions of the fetal GM correspond to those of the adult GM revealed in this study, its structural differences, such as the insertion manner and whole shape, are presumably attributable to the developmental pattern in which the GM arises from two different muscle primordia. Further structural analyses using fetal GM would provide a robust conclusion.

Regarding functional anatomy, the GM is known for extending the hip joint and contributing to other hip joint motions, such as hip abduction and external rotation^6–8^. We found that the superior portion, with a large PCSA, was inserted into the femur via a strong tendon. The distal tendon transitions from the muscle fascicles of the superior portion, these fibers are arranged in series, allowing it to transmit a strong and significant force to the femur. In contrast, the inferior portion is inserted into the complex formed by the distal tendon of the superior portion and the LFIS, with no transitional relation, implying that it indirectly assists the superior portion by tensing its distal tendon. Our findings anatomically corroborate the contribution of the GM as a whole in transmitting force to the femur and its role in hip joint motions, with our detailed evaluation providing fundamental yet further insight.

Nevertheless, there are also several previous anatomical and kinesiological literature that describe the GM contributes to the movement and stabilization of the knee joint via the iliotibial tract^1–3,5,47^. We also found the small accessory fibers of the superior portion that united the iliotibial tract; however, these fibers are presumably unworkable for tibial movement because the iliotibial tract is fixed to the shaft of the femur by the LFIS, as previously described by several authors^14,38,48^. This aligns with kinetic studies by Preece et al. (2008) who found an unclear link between GM activity and tibial rotation during gait^49^and Besomi et al. (2022) who reported no correlation between GM activity and iliotibial tract displacement^15^. Conversely, biomechanical studies of lower limb movement using muscle modeling, which are presented based on previous descriptions of the GM, reported that GM greatly contributes to the movements of both the hip and knee joints as a powerful extensor^50,51^. Based on our precise anatomical data, kinesiological data on the GM should be re-interpreted. This will significantly improve our understanding of human GM function.

## Limitations and perspectives

The division of the GM in present study was not based on the gross anatomy of the intramuscular tendon and also histological analysis; therefore, anatomical verification may not be sufficient. Nevertheless, the macroscopic structural evaluation and findings provide crucial fundamental insight into the functional anatomy of the human GM. However, to fully establish its function, in vivo kinematic analysis is required, which also remains a limitation of this study. Additionally, as this study was conducted on elderly Japanese cadavers, the muscle volume and the ratio between the superior and inferior portions may not be directly applicable to younger individuals or athletes. Future studies incorporating in vivo kinematic or electromyographic analyses could further elucidate the functional anatomy of the human GM and may contribute to advancements in orthopedic medicine, sports science, strength training, and rehabilitation.

## Methods

To clarify the GM structure in cadaveric dissections, we examined 25 sides (five right sides and 20 left sides) of 25 formalin-fixed adult Japanese cadavers (14 males and 11 females). We excluded cadavers with significant pathological alterations in muscle fibers (such as fatty degeneration, intramuscular hematoma, and severe muscle atrophy), traumatic lesions, or operative incisions. All examined cadavers were donated to the Juntendo University School of Medicine for medical education and research, and written informed consent was obtained from the individuals and their families. The study protocol was approved by the Ethics Committee of Juntendo University School of Medicine (Approval No. 2014138). All of the work was carried out in accordance with The Code of Ethics of the World Medical Association (Declaration of Helsinki) for experiments involving humans as revised in 2013, and in consistence with the Japanese law “Act on Body Donation for Medical and Dental Education”.

### Dissection procedures

The structural re-evaluation of the GM was performed using macroscopic dissection and analysis of in situ specimens. The gluteal fascia was completely removed to observe muscle fascicles on the superficial surface of the GM. To observe the muscle fascicles on the deep surface, the proximal part of the GM was detached from the bony pelvis and reflected in its proximal half. In the in situ specimens, all muscles in the gluteal, posterior, and medial thigh regions, except the GM, were removed to confirm where the muscle fascicles and tendinous fibers of the GM were attached.

Additionally, the structures of the proximal and distal ends were re-evaluated by analyzing isolated GM specimens, with reference to findings obtained from in situ dissection to avoid losing information about origin, insertion, and relation to the adjacent structures. The GM was completely isolated from half of the pelvis and thigh as follows: First, the proximal origin of GM was detached from the bony pelvis. The distal end of the GM was then detached from the femur and completely isolated from the thigh, along with surrounding structures such as parts of the iliotibial tract and LFIS, which are associated with the GM distal end (see the Results section). In the isolation process, information about the attachment sites, including origin and insertion, and topographical relations to the adjacent structures, was obtained and appropriately recorded. The nerves and blood vessels of the GM were severed to isolate the GM completely. All findings were recorded using photographs.

To estimate the contribution ratio of the portions of the GM to the total strength of the GM, we calculated the physiological cross-sectional area (PCSA) using the muscle fiber length, muscle weight, and angle of muscle fiber pennation (cos θ) in the muscle compartments of the nine isolated GM specimens following kinesiology texts^52,53^. All muscle fibers of the GM were arranged in a straight line to the tendon and had no pennation angle (cos θ = 1)(refer to Supplementary Fig. 1 and Figs. 1, 2, 3, 4). The length of the muscle fibers was measured using a tape measure. On each superficial and deep aspect of the isolated GM specimens, ten or five locations were determined in the superior or inferior portions, respectively, as equally spaced as possible. At each location, the muscle fiber length was measured from the most proximal to the most distal muscle fiber ends and the average length was calculated separately for superior and inferior portion. Furthermore, the relative PCSA of each GM portion was calculated by considering the total PCSA as 100%. All values are presented as mean ± standard deviation (SD). Differences were tested using Student’s t-tests. Statistical significance was set at *P* < 0.05.

## Electronic supplementary material

Below is the link to the electronic supplementary material.

Supplementary Material 1

Supplementary Material 2

## Data Availability

Upon reasonable request, data supporting the findings of the present study are available from the corresponding authors.
