# Supplementary figures and images for: Structural re-evaluation of the human gluteus maximus
Source: Sci Rep. 2025 Jul 1;15:21251. doi: 10.1038/s41598-025-05361-x (PMC12214855; doi:10.1038/s41598-025-05361-x)

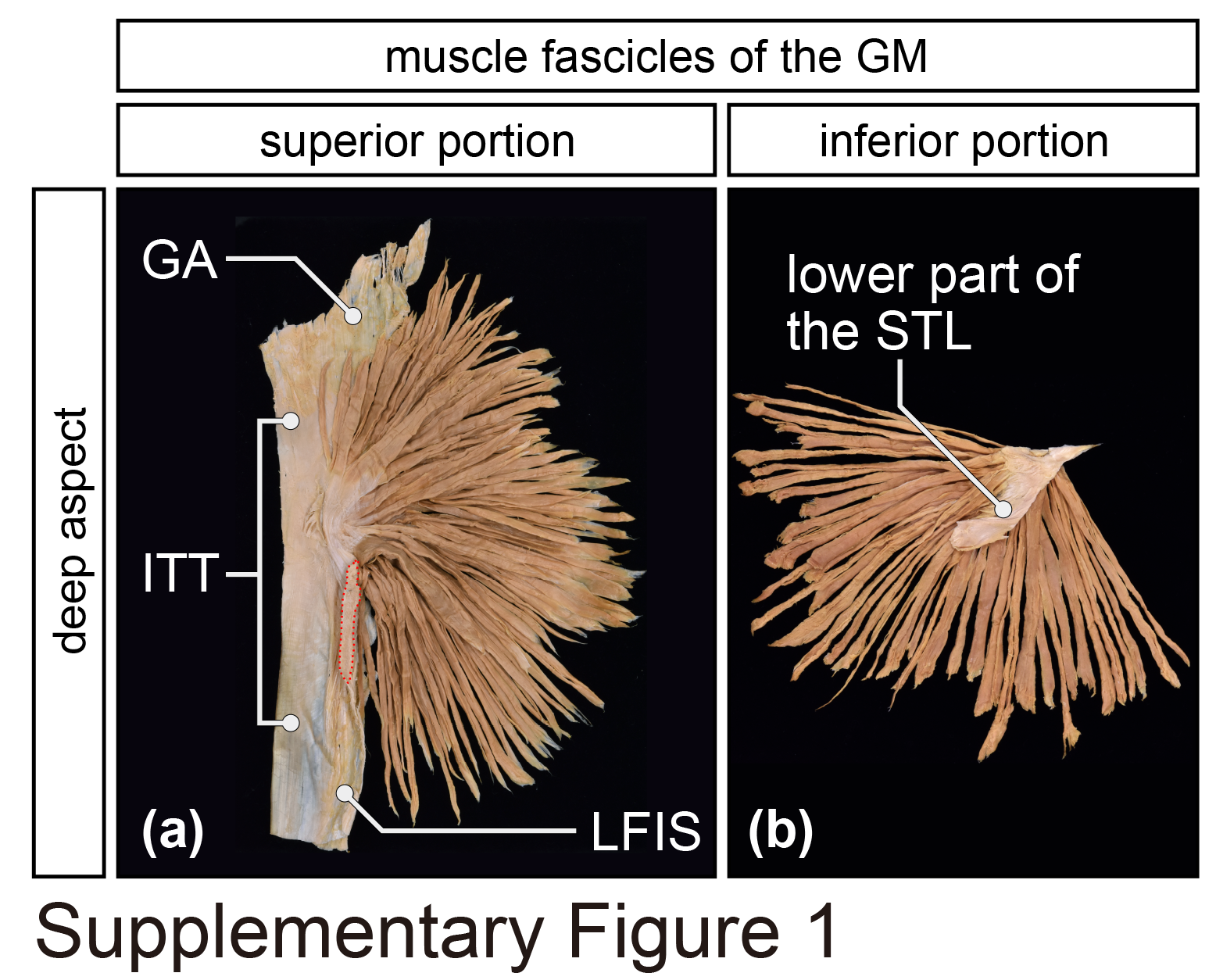

Supplement: Supplementary file 1 — Supplementary Material 1 [file 41598_2025_5361_MOESM1_ESM.tif]
