# Supplementary material for: Structural re-evaluation of the human gluteus maximus
Source: Sci Rep. 2025 Jul 1;15:21251. doi: 10.1038/s41598-025-05361-x (PMC12214855; doi:10.1038/s41598-025-05361-x)
Supplement: Supplementary file 2 — Supplementary Material 2 [file 41598_2025_5361_MOESM2_ESM.docx]

**Supplementary information**

**Figure S1. Muscle fascicles of the superior and inferior portions of the GM**

The numerous thick muscle fascicles of the gluteus maximus (GM) are shown by removing the fascia and isolating the fascicles individually. (a) and (b) show the superior and inferior portions from their deep aspects, respectively. GA, gluteal aponeurosis; GM, gluteus maximus; ITT, iliotibial tract; LFIS, lateral femoral intermuscular septum; STL, sacrotuberous ligament.
